# Bisphenol A and Ovarian Reserve among Infertile Women with Polycystic Ovarian Syndrome

**DOI:** 10.3390/ijerph14010018

**Published:** 2016-12-27

**Authors:** Wei Zhou, Fang Fang, Wenting Zhu, Zi-Jiang Chen, Yanzhi Du, Jun Zhang

**Affiliations:** 1 Ministry of Education-Shanghai Key Laboratory of Children’s Environmental Health, Xinhua Hospital, School of Medicine, Shanghai Jiao-Tong University, Shanghai 200092, China; zzsmile12@163.com (W.Zhou); fangfang19881106@163.com (F.F.); windy5490@163.com (W.Zhu); 2 Center for Reproductive Medicine, Shandong Provincial Hospital, Shandong University, Shandong 250100, China; chenzijiang@vip.163.com; 3 Ministry of Education-The Key Laboratory of Reproductive Endocrinology, Shandong University, Shandong 250100, China; 4 Center for Reproductive Medicine, Ren Ji Hospital, School of Medicine, Shanghai Jiao Tong University, Shanghai 200092, China; yanzhidu@hotmail.com; 5 Shanghai Key Laboratory for Assisted Reproduction and Reproductive Genetics, Shanghai 200092, China

**Keywords:** bisphenol A, ovarian reserve, antral follicle count, antimullerian hormone, day-3 follicle stimulating hormone

## Abstract

To better understand possible effects of bisphenol A (BPA) exposure on ovarian reserve in women with polycystic ovary syndrome (PCOS), we measured creatinine adjusted urinary BPA (BPA_Cre) concentrations and used regression models to evaluate the association between urinary BPA level and antral follicle count (AFC), antimullerian hormone (AMH), day-3 follicle stimulating hormone levels (FSH) and inhibin B (INHB) in 268 infertile women diagnosed with PCOS. BPA was detected in all women with a median concentration of 2.35 ng/mL (the 25th and 75th percentiles of 1.47 ng/mL and 3.95 ng/mL). A unit increase in BPA_Cre was associated with a significant decrease of 0.34 in AFC (β = −0.34, 95% CI = −0.60, −0.08; *p* = 0.01). Likewise, BPA was negatively associated with AMH and day-3 FSH levels, but neither of them reached statistical significance. No association was observed between BPA and INHB. Our results suggest that in women with PCOS, BPA may affect ovarian follicles and, therefore, reduce ovarian reserve.

## 1. Introduction

Evidence seems to suggest that human fertility has been declining over the past few decades [1]. Since genomics are relatively stable over a short time period at the population level, lifestyle-related and environmental factors may play an important role in the declining human fertility [2].

Endocrine disrupting chemicals (EDCs) are natural or synthetic chemical compounds that can interfere with the endocrine system by mimicking or antagonizing endogenous steroid hormones. Bisphenol A (BPA) is widely used in the manufacture of resins such as polycarbonate plastic products and epoxy resins [3]. More than 3.5 million tons of BPA are produced a year worldwide and over 100 tons are released into the atmosphere [4]. The main routes of daily exposure are ingestion, inhalation and dermal absorption [2].

BPA has estrogenic activity and can bind to α- and β-estrogen receptors (ER) both in vivo and in vitro [3]. It has been detected in almost all human body fluids, including follicular fluid [5], which indicates that oocytes are exposed to BPA during the folliculogenesis process [6]. A large number of animal experiments suggest that BPA may act as a reproductive toxicant and affect fertilization rate, number of live newborns per litter, the onset of female puberty and estrous cycle [7,8]. Moreover, animal studies indicate that BPA has adverse effects on the maturing oocyte and meiotic cell division machinery [9]. In women undergoing in vitro fertilization (IVF) treatment, an inverse association was found between urinary BPA concentrations and oocytes maturation, number of oocytes retrieved and peak estradiol (E2) levels [10]. These findings raised concerns that environmental BPA exposure may adversely affect the oocyte quality and cause the decline of ovarian reserve and fertility in the general population, though the magnitude of actual risk in human remains uncertain [7].

Polycystic ovarian syndrome (PCOS) is the most common endocrine disorder among reproductive age women, affecting up to 5% to 10% of women [11], This disorder is characterized by oligo/anovulation, high androgen level and often accompanied by infertility. Several studies have reported a higher BPA level in women with PCOS than in regularly ovulating women [12,13]. However, it remains poorly understood whether BPA may affect ovarian reserve and function in these women. Findings of this study may help us elucidate how BPA may reduce ovarian function in women with PCOS.

## 2. Materials and Methods

### 2.1. Study Participants

Between June and October 2014, 268 infertile women due to PCOS were recruited in our study at the Center for Reproductive Medicine, Shandong University. Only women who never had any hormone treatment, including oral contraceptives, or other relevant medications before having the test of ovarian reserve were eligible. Women who had previous ovarian surgery were excluded due to a possible effect of the surgical intervention on the total antral follicle count (AFC). PCOS was diagnosed based on the Rotterdam criteria (Rotterdam ESHRE/ASRM-Sponsored PCOS Consensus Workshop Group, 2004), including at least two of the following symptoms: oligo/anovulation, biochemical and/or clinical hyperandrogenism and polycystic ovaries on ultrasound, with other androgen excess disorders excluded [14]. The study was approved by the Ethics Committee of the Women’s Hospital, School of Medicine, Zhejiang University (20130044) and all subjects gave their informed consent for inclusion before they participated in the study.

### 2.2. Urine Sample Collection and Urinary BPA Concentrations Measure

About 100 mL urine sample was collected in a sterile polypropylene cup from each subject, aliquoted to polypropylene storage tubes (15 mL, 430791 CentriStar, Corning, CA, USA) and stored at –20 °C prior to BPA analysis. Urinary BPA concentration was measured using high performance liquid chromatography-electrospray ionization tandem mass spectrometry (HPLC-MS/MS) (Agilent 1290-6490, Agilent Technologies, Little Falls, DE, USA). The limit of detection (LOD) was 0.1 µg/L. All the intra- and inter-batch precisions were less than 15%. BPA concentrations were corrected for urine dilution by creatinine (Cre). Cre concentration was measured using enzymatic method on automatic chemical analyzer (7100 Automatic Analyzer, Hitachi, Tokyo, Japan). The detailed description of the analytical method is provided elsewhere [15].

AFC was determined by transvaginal ultrasound in the reproductive center. All ultrasound exams were performed on the 2nd or 3rd day of the menstrual cycle. No fertility treatments were used before the AFC determination. Day-3 serum follicle stimulating hormone (FSH), antimullerian hormone (AMH), inhibin B (INHB) were measured at the reproductive center using an electrochemiluminescence immunoassay (for FSH, Roche Diagnostics, Basel, Switzerland), an enzyme-linked immunosorbent assay (for AMH and INHB, Ansh Labs, Webster, TX, USA), respectively.

### 2.3. Statistical Analysis

Women’s characteristics, BPA concentration and outcomes of interest were summarized using median and percentages as appropriate. Multivariable linear regression models were applied to evaluate the association between BPA_Cre and AFC, AMH, FSH and INHB levels. Factors that may affect the relationship between BPA level and ovarian reserve predictors were chosen as potential confounders. Covariates, including age, BMI, and household income, were considered to be confounders in the regression model as they changed the point estimates of interest by greater than 10%. Data analysis was performed using SAS version 9.2 (SAS Institute Inc., Cary, NC, USA) and a *p*-value of <0.05 was considered to be statistically significant. Smoothing spline was applied to depict the relation of BPA exposure and ovarian reserve predictors using EmpowerStats software (X & Y Solutions Inc., Boston, MA, USA).

## 3. Results

Urinary BPA concentrations were measured in all 268 women, but not all tests were done in all women: AFC, AMH, FSH, and INHB results were available from 215, 257, 250 and 131 women, respectively. A total of 104 women were available for these four tests. Women’s characteristics of the sample population are shown in Table 1. The medians of urinary BPA concentrations were virtually the same among women included in the subdatasets (Table 1). All samples had BPA concentration above the LOD (0.1 ug/mL) with the median of 2.35 ng/mL and the 25th and 75th percentiles of 1.47 ng/mL and 3.95 ng/mL.

**Figure 1:**
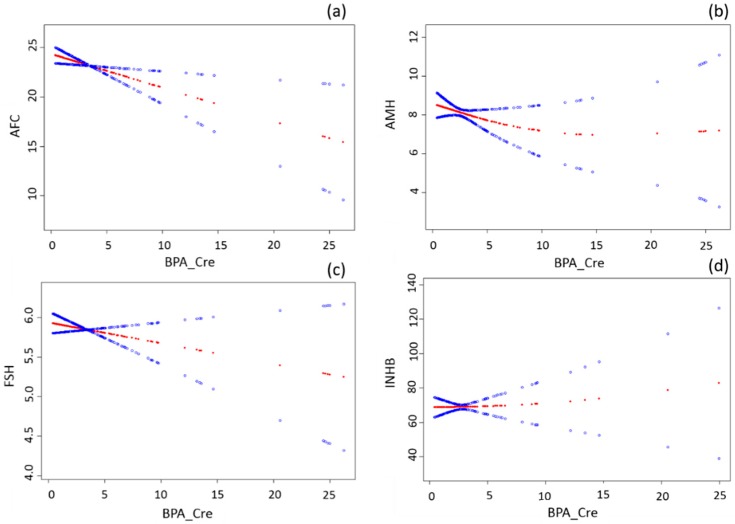
The relationships between BPA and antral follicle count, antimullerian hormone, inhibin B, and follicle stimulating hormone levels. The x-axis refers to urinary BPA level corrected by creatinine concentration (BPA_Cre). The y-axis refers to AFC (**a**); AMH (**b**); FSH (**c**); and INHB (**d**) levels. The red line is fitted by generalized additive model showing the relationship between x and y axes. The two blue lines refer to 95% confidence intervals. All models were adjusted for age, BMI, income.

Figure 1 illustrates the relationships between BPA and the follicular reserve parameters. Multivariable Linear regression models suggested an inverse association between urinary BPA concentration and AFC (Table 2). In the crude model, a unit increase in BPA_Cre was associated with a significant decrease of 0.32 in AFC (β = −0.32, 95% CI = −0.57, −0.07; *p* = 0.01). The association was similar in the adjusted model (β = −0.34, 95% CI = −0.60, −0.08; *p* = 0.01) after controlling for age, BMI, and household income (Table 2). Likewise, BPA was negatively associated with AMH and day-3 FSH levels but neither of them reached statistical significance. BPA was not associated with INHB.

## 4. Discussion

We found that BPA could be detected in all women. There was a significant inverse association between urinary BPA concentration and AFC in women with PCOS, which is consistent with a previous study with 154 women undergoing infertility treatments [5]. Although statistically nonsignificant, BPA was negatively associated with AMH and day-3 FSH. These findings suggest that environmental BPA exposure may have negative impact on ovarian reserve.

The growth and maturation of oocytes are crucial processes of gamete development. Many steroids, genes and related proteins are involved in the process of oogenesis and maturation [16]. Experimental studies have shown that BPA can directly affect oocyte or indirectly affect the local, intra-ovarian and intrafollicular environment (i.e., by affecting the granulosa cells that are essential for ovarian follicle growth, steroidogenesis, oocyte survival and nourishment) [2]. For example, rodents exposed to BPA during the early postnatal period had a reduced ovarian follicular reserve, including a decline in the stock of primordial follicles, increase in antral atretic follicles, higher incidence of multiple oocyte follicles (MOFs) and lower ovarian weight [16,17]. The increased incidence of MOFs may also contribute to the decline in the primordial follicle pool. These findings were confirmed in the lamb, a precocial species in which the follicular development trajectory is similar to humans [18].

BPA is commonly described as a “weak” estrogen that binds to the nuclear ERβ with approximately seven times greater affinity than to ERα [18]. Jefferson et al. [19] found that ERβ-knockout mice exposed to estrogenic compounds didn’t form MOFs, while wild-type or ERα-knockout mice did. Thus, the effects of estrogen on early folliculogenesis and follicle development may be mediated through ERβ [18].

One study [20] showed that BPA exposure during pre-puberty period reduced ovarian weights and follicle numbers and increased the constituent ratio of atretic follicles in Wistar rats. The effect may be caused by the decreased expression of follicle development-related genes such as FIGLA, H1FOO with increasing BPA doses. Compared with the control group, the expression of FIGLA gene mRNA and protein were both significantly reduced in the highest BPA group (160 mg/kg/day). However, H1FOO gene mRNA and protein expression levels showed a significant decrease in all three BPA groups (10/40/160 mg/kg). The adverse effect was consistent with our human study.

Epigenetic alterations induced by BPA exposure were also the focus in other studies [16,21]. Chao et al. investigated the effects of BPA on DNA methylation of imprinted genes and its potential influence on oocyte development in mice [16]. They demonstrated that BPA exposure during early postnatal period resulted in remarkably decreased methylation of imprinted gene IGF2R and PEG3 and suppressed expression of DNA methylation transferases (Dnmts) which were closely related to oocyte growth. In addition, BPA accelerated the depletion of the primordial follicle pool, suppressed the meiotic maturation of oocytes because of abnormal spindle assembling in meiosis, and enhanced estrogen receptor (ER) expression at the levels of mRNA and protein. With an ER inhibitor, the methylation process of the imprinted gene and the mRNA expression of Dnmts were recovered. Thus, BPA may suppress methylation of imprinted gene by affecting ER expression. Dolinoy et al. [21] also demonstrated that BPA decreased CpG (cytosine-guanine dinucleotide) methylation of an intracisternal A particle retrotransposon upstream of the Agouti gene and CDK5 activator-binding protein (CabpIAP), indicating that BPA may affect oocyte maturation via direct or indirect epigenetic modification of related genes.

It is well known that granulosa cells play a pivotal role in ovarian physiology through producing steroid hormones and other factors during the follicular development [22]. The disruption of their functions induced by BPA could have a significant impact on folliculogenesis. Some studies [23,24] documented that murine ovarian granulosa cells cultured with BPA had increased granulosa cell apoptosis, decreased granulosa cell viability and increased follicular atresia. Sex steroid hormone production is essential for the development of antral follicles. Both in vivo and in vitro studies have demonstrated that higher BPA levels is associated with decreased estradiol (E2) synthetized in ovarian [25,26]. The impaired steroid biosynthesis induced by BPA may also affect folliculogenesis process.

To the best of our knowledge, this is the largest human study that evaluates the associations between environmental exposure of BPA and ovarian reserve. However, several limitations are worth mentioning. First, misclassification of BPA exposure due to BPA short half-life in human and variability of the exposure over time is possible because a single urine sample may not be representative of the overall exposure of an individual. This misclassification is likely to be non-differential, which may have drawn our conclusions towards the null. On the other hand, we speculate that lifestyle habits will not change dramatically over short time intervals and the spot urinary BPA concentration may reflect the general exposure level of an individual to some degree. Second, since this is a cross-sectional study, a causal inference may not be made between BPA and ovarian reserve. Finally, it is uncertain whether our findings can be generalized to the general population because our volunteers were diagnosed infertile patients with PCOS.

## 5. Conclusions

In this cross-sectional study, an association between environmental exposure to BPA and decreased AFC was observed among women with PCOS, suggesting that BPA may affect human ovarian function. More studies are needed to confirm our findings in the general population and further elucidate the complex mechanisms through which BPA affect the reproductive health of women.

## Figures and Tables

**Table 1:** Demographic characteristics and measures of ovarian reserve.

Characteristic	AFC (*n* = 215)	AMH (*n* = 257)	FSH (*n* = 250)	INHB (*n* = 131)
Age (years)	27 (25, 31)	27 (25, 30)	27 (25, 30)	28 (26, 32)
BMI (kg/m^2^)	25.4 (22.5, 27.7)	25.4 (22.1, 28.2)	25.4 (22.1, 28.3)	25.4 (22.0, 28.3)
Income (10^3^ RMB/person/year) (*n*, (%))				
<10	43 (20)	53 (21)	52 (21)	22 (17)
10–30	112 (52)	138 (54)	134 (54)	66 (50)
30–50	32 (15)	37 (14)	35 (14)	25 (19)
50–100	14 (6)	13 (6)	14 (6)	9 (7)
>100	6 (3)	6 (2)	6 (2)	5 (4)
Refuse to answer	8 (4)	9 (3)	9 (3)	4 (3)
BPA (ng/mL)	2.3 (1.1, 3.8)	2.1 (1.1, 3.8)	2.1 (1.1, 3.8)	2.3 (1.1, 3.7)
BPA_Cre (ng/g)	2.2 (1.4, 3.9)	2.2 (1.4, 3.7)	2.2 (1.4, 3.9)	2.2 (1.3, 3.5)
AFC	23 (18, 28)			
AMH (ng/mL)		6.7 (4.1, 11.7)		
FSH (IU/L)			5.8 (5.1, 6.6)	
INHB (pg/mL)				66.2 (42.7, 87.8)

Data are expressed as median (25%, 75% percentile); BMI: body mass index; AFC: antral follicle count; AMH: antimullerian hormone; FSH: follicle stimulating hormone; INHB: inhibin B; BPA: bisphenol A.

**Table 2:** The association of urinary BPA levels **^a^** with antral follicle count, antimullerian hormone, day-3 follicle stimulating hormone and inhibin B levels.

Indicators	*n*	Crude β (95% CI)	*p*-Value	Adjusted β (95% CI) ^b^	*p*-Value
AFC	215	−0.32 (−0.57, −0.07)	0.01	−0.34 (−0.60, −0.08)	0.01
AMH	257	−0.06 (−0.21, 0.08)	0.39	−0.08 (−0.22, 0.07)	0.30
FSH	250	−0.02 (−0.06, 0.02)	0.23	−0.03 (−0.07, 0.01)	0.20
INHB	131	0.48 (−1.30, 2.27)	0.59	0.42 (−1.41, 2.25)	0.65

**^a^** BPA_Cre as a continuous variable; **^b^** Multivariable linear regression adjusting for age, BMI and Income.
